# Clinical, epidemiological aspects, and trends of Hepatitis B in Brazil from 2007 to 2018

**DOI:** 10.1038/s41598-021-93434-y

**Published:** 2021-07-07

**Authors:** Cathianne Sacramento Pinto, Galileu Barbosa Costa, Ivan Bezerra Allaman, Sandra Rocha Gadelha

**Affiliations:** 1 Departamento de Ciências da Saúde, Universidade Estadual de Santa Cruz, Ilhéus, Bahia Brazil; 2 Departamento de Ciências Exatas e Tecnológicas, Universidade Estadual de Santa Cruz, Ilhéus, Bahia Brazil; 3 Departamento de Ciências Biológicas, Universidade Estadual de Santa Cruz, Ilhéus, Bahia Brazil

**Keywords:** Diseases, Health care, Risk factors, Virology, Hepatitis B virus, Viral epidemiology, Epidemiology

## Abstract

Hepatitis B virus (HBV) infection is a concern for public health due to its high prevalence, high infectivity, morbidity, and mortality worldwide. Brazil presents a low HBV prevalence, but has considerable heterogeneity among its geographic regions. Here, we describe the epidemiological profile of HBV infection in different regions of Brazil during 2007–2018, as well as the historical trends associated with the infection. We conducted an observational, ecological time-series study using secondary data collected from the National Notifiable Diseases Information System (SINAN). Our findings suggest that HBV infection was more likely to occur in young, sexually active adults. Individuals from Northeast and Midwest regions were more likely to present acute HBV infection, while individuals from South region were more likely to present chronic HBV infection, reinforcing that specific strategies are required for each particular region. Additionally, we observed a general decreasing trend of infection starting in 2014, however there was an increasing trend of infection in men and in individuals over 40 years old. Although we observed a decreasing trend in HBV infection, active surveillance is needed to prevent HBV spread and possible epidemics, as well as encouraging the vaccination of adults, especially young adult males. Our findings can inform the conduct of large-scale observational studies to evaluate clinical, economical, and social impacts of HBV infections, leading to improved social policies. Finally, our results highlight the need to improve data quality and completeness of epidemiological data, minimizing eventual errors that can make prevention and control strategies difficult.

Hepatitis B virus (HBV) infection is considered a burden to public health worldwide due to the chronic nature of infection and the risks of cirrhosis and liver cancer^1,2^. It is estimated that more than 300 million people are chronically infected with HBV, being most infections occurring in developing countries^1^. The prevalence of HBV infection in Brazil, approximately 6,7/100.000 inhabitants, is considered low, but it is heterogeneous throughout country^3^. Brazil is one of the most populous countries in the world, with a vast territory subdivided into five geographic regions: North, Northeast, Midwest, Southeast and South. These regions differ in economic, social and cultural aspects, which directly impact the health conditions of the population, producing multiple scenarios for health, education and income indicators^4^. In addition, within the same area there may also be discrepancies regarding HBV infection, such as higher prevalence in some specific populations, such as homeless people^5^, HIV-positive, individuals undergoing hemodialysis or coagulopathy treatment^6^ and resident of rural areas^7^.

Unprotected sex and sharing contaminated fomites are important recognized risk factors associated with HBV infection^1^. HBV can be found in body fluids such as blood, serum, saliva, nasopharyngeal, and urine of chronically infected individuals at significant amounts (up to 10^9^ copies/ml)^8^, and viral particles are very resistant, surviving at room temperature on dry surfaces for up to 30 days^2^. The clinical course of HBV infection varies considerably from asymptomatic to acute or chronic disease, also evolving to serious fulminant complications such as cirrhosis and hepatocellular carcinoma^1,2^. HBV infection is also associated with multiple non-hepatocellular cancers including non-Hodgkin's lymphoma, cholangiocarcinoma and, pancreatic cancer besides biliary, cervical, uterine, breast, thyroid, lung and skin cancers^9,10^.

Efforts have been made to enhance the prevention and treatment of HBV infection worldwide. The availability of a vaccine, recommended for individuals up to 18 years old and for those at high risk for infection, as well as through therapies based on the administration of interferon and nucleos(t)ide analogues, are effective strategies applied to successfully control HBV transmission and infection^11^. In Brazil, the Brazilian Ministry of Health (BMS) has adopted different strategies to reduce HBV transmission, such as rapid diagnostic tests, post-exposure chemoprophylaxis, free condom distribution, and educational campaigns on HBV infection counseling^3^. HBV vaccination has had its target population gradually expanded and is currently offered indiscriminately and free of charge in public facilities^1,3^. In addition, viral hepatitis has been on the list of Compulsory Notification Disorders since 1996, in which confirmed cases must be reported within 7 days through the National Notifiable Diseases Information System (SINAN) Viral Hepatitis reporting form^3^. The epidemiological profile, as well as the trend of HBV infection in Brazil are still poor explored, having a gap in at least the last 11 years. Therefore, we aimed to evaluate the profile of HBV infection throughout Brazil during 2007–2018, as well as to evaluate the trends associated with HBV infection. In addition, this study represents an opportunity to discuss the impact of underreporting cases of HBV infection and the importance of completeness of data.

## Methods

An observational, ecological time-series study was conducted using secondary data collected from the SINAN, available through the Informatics Department of the National Health System (DATASUS/TABNET), which is part of the Brazilian Ministry of Health (http://www2.datasus.gov.br/DATASUS/index.php?area=02)^12^. SINAN is available in all Brazilian municipalities and states, providing data, evaluation, and monitoring of actions related to diseases and conditions on the national list of nationally notifiable diseases^13^. Furthermore, the secondary data sources available at SINAN can provides potential applications to generate strong interventions and policy decisions. The cases and data notified by SINAN are collected at the health facilities through epidemiological disease surveillance reporting forms^12^.

A confirmed case of HBV provided by the SINAN database is defined according to the presence of one or more criteria as follows: (1) detection of HBV antigen (HBsAg), which is performed mainly through rapid tests; (2) confirmation of other HBV markers such as the presence of viral DNA through real-time or conventional PCR; and (3) the quantification of viral load. It is important to say that the definition criteria were established by the Ministry of Health and not by our research group. All the findings reported in our manuscript are legitimate and reflects what is available in the DATASUS database. Our research group was not involved in any of the activities that were responsible for collecting the clinical and epidemiological information on the field.

Notifications of HBV infection confirmed by clinical and laboratory criteria from 2007 to 2018 from all Brazilian regions were included together with variables such as gender, age, and presumptive source of infection. It is important to emphasize that Brazil is divided into five large geographic regions (North, Northeast, Central-West, Southeast, and South), according to natural, cultural, social, political, and economic similarities^14^.

Fields marked as ignored or blanks, as well as results from foreign individuals without permanent residence in Brazil were excluded. Statistical analysis for databases with more than 2 rows and 2 columns was performed using Chi-squared test to evaluate the hypothesis of independence among the HBV infection and different exposition factors, with a significance level of 5% (*p* ≤ 0.05) applied. Once the independence hypothesis was rejected, the correspondence analysis was applied in order to understand which row variables were related to the column variables. The correspondence analysis is a very robust statistical technique as inference techniques such as Newman-Pearson are, and is very recommended to studies similar to the proposed one^15–18^.

For p-row and 2-column databases it was used loglinear model analysis. The piecewise regression technique (also known as Joinpoint regression or segmented regression) was used because it fits the points better than traditional polynomial regression. The break points chosen in the tendency analysis were 2011 and 2014, and then the linear–linear–linear piecewise regression was considered, i.e., there were three equations of the first degree for the passages cited for each region (2007–2011; 2012–2014; 2015–2018). Since the interest was verified if the trends between regions differ from each other, we evaluated the significance of each parameter and the confidence interval for the slope coefficient to compare which intervals were overlapping between regions. The linear regression parameter statistics were performed for each region. The b1, b2, and b3 was considered as estimators for the periods from 2007 to 2011, from 2012 to 2014, and from 2015 to 2018, respectively. Analysis was performed on R software^19^ with the aid of the ca function of the ca package^20^ version 0.71 for the correspondence and glm function analysis of the stats package^21^ version 3.5.3.

This study analyzed secondary data collected from a public domain database (SINAN), thus no direct consent from study participants was needed. All analyses were conducted in accordance with good clinical practices.

## Results

During 2007–2018 a total of 172,090 cases of HBV infection were reported by SINAN. Supplementary table S1 shows the distribution of HBV cases per region of Brazil. Most cases (33.9%) were reported in Southeast region, while the Midwest region reported the lower number of cases (9.0%). The greatest number of cases were reported during 2013 (10.2%) and 2014 (10.3%).

Demographic characteristics of the patients are presented in Table 1. Most individuals were male (54.4%) and the most common education level was completion of secondary education (32.3%). Illiteracy was reported by 1.8% of individuals only. Individuals from 0 to 9 years old presented a low proportion (2.0%) of the total cases of HBV infection, while a higher proportion was observed among adults (from 20 to 79 years old, 41.0%) (Table 2). Table 3 displays the clinical characteristics of HBV infection according to gender, age, and region. Chronic hepatitis B accounted for the majority of cases, followed by acute and fulminant clinical forms. A total of 6944 cases were inconclusive. Information regarding asymptomatic cases and missing data per regions of Brazil were not available. Supplementary table S3 shows the distribution of clinical forms of HBV infection during the years. It is also important to emphasize that there are relevant missing data for all the variables listed above (Supplementary table S3).

**Table 1:** Demographic characteristics of individuals affected by Hepatitis B virus infection in Brazil during 2007–2018.

Demographics	HBV confirmed cases (N = 172,090)	%
**Gender**		
Female	78,355	45.5
Male	93,710	54.4
Missing data	25	0.01
**Age (years)**		
< 1	831	0.5
2–4	309	0.2
5–9	534	0.3
10–14	1160	0.7
15–19	6408	3.7
20–39	77,737	45.2
40–59	74,501	43.3
60–79	9519	5.5
≥ 80	1055	0.6
Missing data	33	0.02
**Education**		
Primary	49,524	28.8
Secondary	55,525	32.3
Higher	12,889	7.5
Illiteracy	3176	1.8
Missing data	50,976	29.6

**Table 2:** Proportion of presumptive sources of infection for Hepatitis B virus according to age in individuals from Brazil during 2007–2018.

Presumptive source of infection	Children 0–9 years n (%)^a^	Adolescents 10–19 years n (%)^a^	Adults 20–59 years n (%)^a^	Elderly ≥ 60 years n (%)^a^
Sexual	290 (15.0)	1995 (25.7)	37,942 (26.4)	3227 (17.7)
Transfusion	42 (2.2)	110 (1.4)	2917 (2.1)	731 (4.0)
Intravenous drug use	23 (1.2)	98 (1.3)	3367 (2.3)	128 (0.7)
Vertical	345 (17.8)	661 (8.5)	3947 (2.7)	276 (1.5)
Occupational accident	4 (0.2)	9 (0.1)	552 (0.4)	42 (0.2)
Hemodialysis	6 (0.3)	14 (0.2)	353 (0.2)	107 (0.6)
Household contact	162 (8.4)	634 (8.3)	5459 (3.8)	516 (2.8)
Surgical treatment	19 (1.0)	38 (0.5)	1994 (1.4)	595 (3.2)
Dental treatment	71 (3.7)	248 (3.2)	4160 (2.9)	619 (3.4)
Missing data	969 (50.2)	3961 (51.0)	82,795 (57.7)	11,975 (65.7)
Total individuals	1931	7768	143,486	18,216

^a^The percent values are represented by column.

**Table 3:** Clinical characteristics of Hepatitis B infection in Brazil during 2007–2018.

Variables	Total	Acute	Chronic	Fulminant	Inconclusive diagnosis	Clinical diagnosis not informed
**Gender**						
Female	82,127	10,189 (12.4%)	66,932 (81.5%)	91 (0.1%)	3336 (4.0%)	1579 (2.0%)
Male	98,946	12,866 (13.0%)	80,379 (81.2%)	203 (0.2%)	3607 (3.6%)	1891 (1.9%)
Missing	26	2 (7.7%)	21 (80.7%)	1 (3.8%)	1 (3.8%)	1 (3.8%)
**Age (years)**						
Children (0–9)	2121	487 (23.0%)	1492 (70.3%)	4 (0.2%)	88 (4.1%)	50 (2.3%)
Adolescents (10–19)	8362	1412 (16.9%)	6413 (76.7%)	12 (0.1%)	330 (3.9%)	195 (2.3%)
Adults (20–59)	151,377	19,069 (12.6%)	123,373 (81.5%)	230 (0.1%)	5824 (3.8%)	2881 (1.9%)
Elderly (≥ 60)	19,201	2083 (10.8%)	16,023 (83.4%)	49 (0.2%)	701 (3.6%)	345 (1.8%)
Missing	35	6 (17.1%)	28 (80.0%)	0	1 (2.8%)	0
**Region of Brazil**^**a**^						
North	27,490	3647 (13.3%)	23,136 (84.1%)	32 (0.1%)	675 (2.4%)	NA
Northeast	18,035	3837 (21.2%)	13,300 (73.7%)	33 (0.2%)	865 (4.8%)	NA
Midwest	60,661	3048 (19.3%)	11,986 (76.0%)	16 (0.1%)	716 (4.5%)	NA
South	55,676	4822 (8.7%)	48,662 (87.4%)	99 (0.2%)	2093 (3.7%)	NA
Southeast	15,766	7703 (12.7%)	50,248 (82.8%)	115 (0.2%)	2595 (4.3%)	NA

^a^Information on missing cases related to clinical HBV forms per regions of Brazil was not available. *NA* not available.

**Figure 1:**
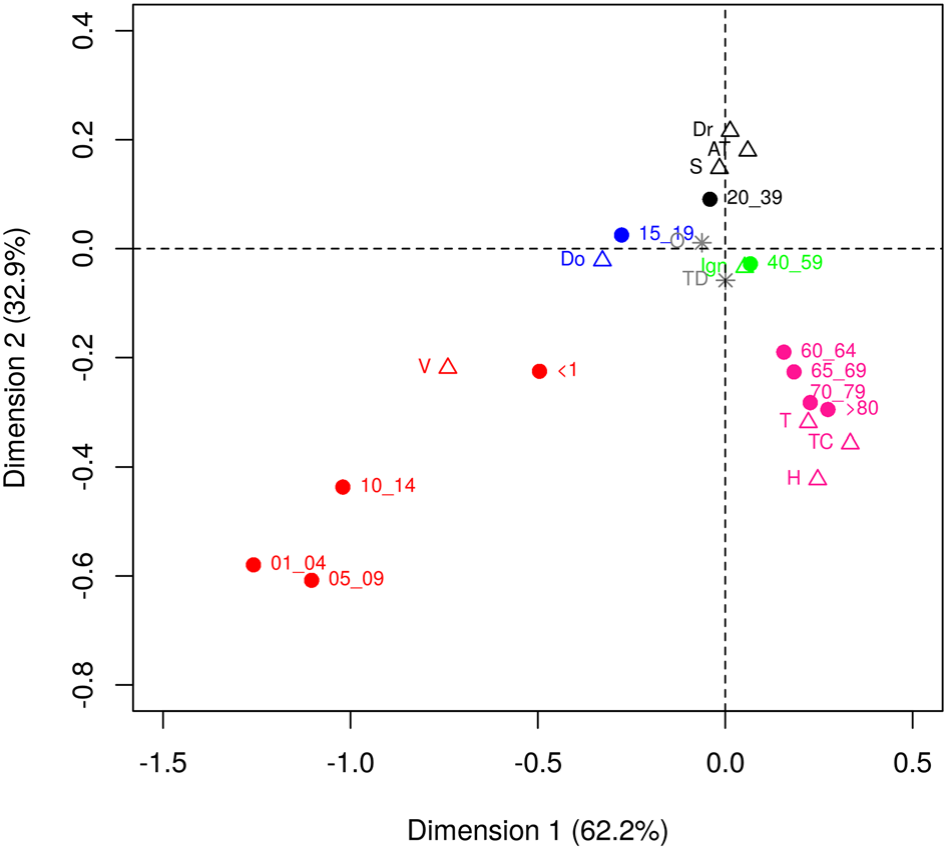
Map of the relationship between age and presumptive source of infection. Age (years) was categorized as < 1; 1–4; 5–9; 10–14; 15–19; 20–39; 40–59; 60–64; 65–69; 70–79; > 80. Presumptive sources of infection were coded as Sexual (S); Transfusion (T); Intravenous drug use (Dr), Vertical (V), Occupational Accident (AT), Hemodialysis (H), Household Contact (Do), Surgical Treatment (CT), and Dental Treatment (TD). This figure was created by using the R software^19^ version 3.5.3 (https://www.R-project.org/).

Regarding the sources of infection, significant differences were observed between the age groups ($\ddot{y}^{2}$ = 7083,88; DF = 100; *P* ≤ 0.0001) as shown in Fig. 1. Individuals up to 19 years old were more likely to be infected by close contact with infected individuals in their household and during vertical transmission. We also found that individuals from Northeast and Midwest regions were more likely to present acute HBV infection, while individuals from South region were more likely to present chronic HBV infection (Table 4). No association with any specific clinical form was observed in individuals from North region. Regarding presumptive source of infection, no associations were observed for individuals from Northeast and Midwest regions. However, there was a strong association with sexual contact for individuals from North region.

**Table 4:** Characteristics associated with HBV infection in different regions of Brazil from 2007 to 2018, according to the correspondence analysis.

Region	Main associated features		
	Clinical form	Presumptive source of infection	Age (years old)
North	No association^a^	Sex	0–39
Northeast	Acute	No association^a^	0–39
South	Chronic	Transfusion	40–79
		Vertical transmission	
		Surgical and dental treatment	
Southeast	No association^a^	No association^a^	40–79
Midwest	Acute	No association^a^	0–39

^a^There was no strong association with at least one source of infection specifically.

**Figure 2:**
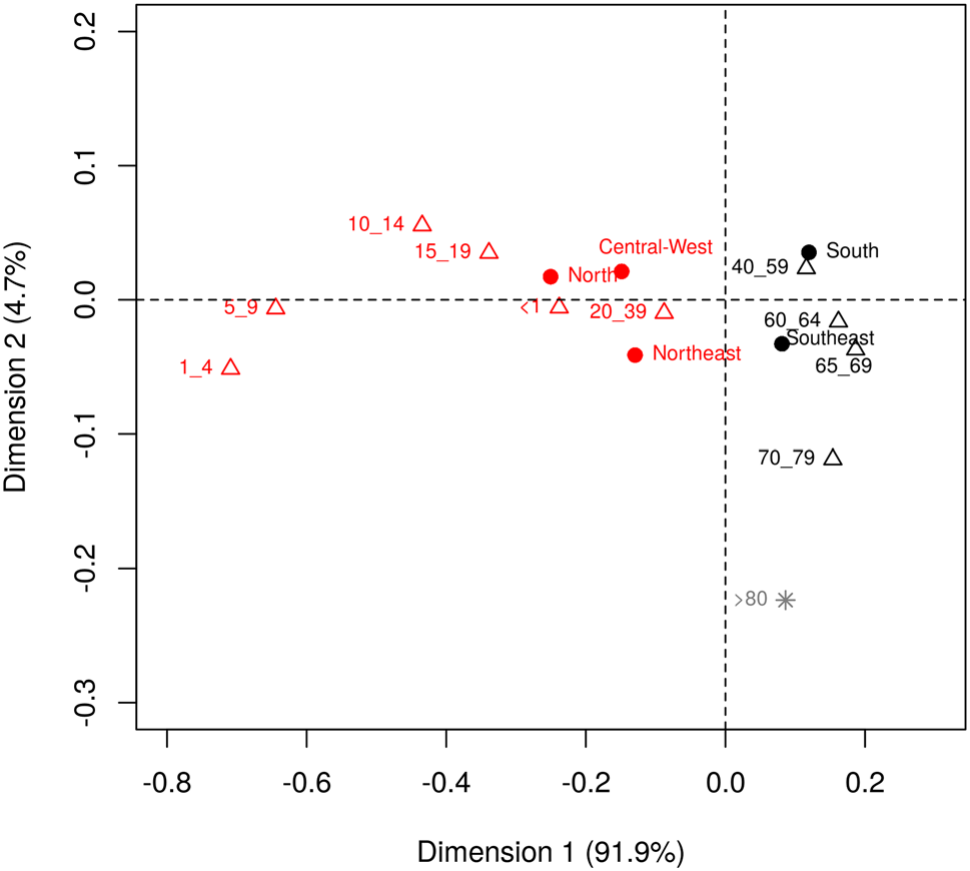
Relationship between age of hepatitis B carriers and regions of Brazil from 2007 to 2018. Age (years) was categorized as < 1; 1–4; 5–9; 10–14; 15–19; 20–39; 40–59; 60–64; 65–69; 70–79; > 80. The five regions of Brazil are represented as North (norte), Northeast (Nord), Midwest (C_O), South (Sul) and Southeast (Sud). This figure was created by using the R software^19^ version 3.5.3 (https://www.R-project.org/).

The most affected age groups by region are shown in Fig. 2. It can be observed a contrast between the North, Midwest, and Northeast regions with the South and Southeast regions. Besides, there is a contrast between the two age groups: 0 to 39 years old and 40 to 79 years old. The 0–39 years old group were more associated with the North, Midwest and Northeast regions, while the 40–79 years old group were more associated with the South and Southeast regions.

**Figure 3:**
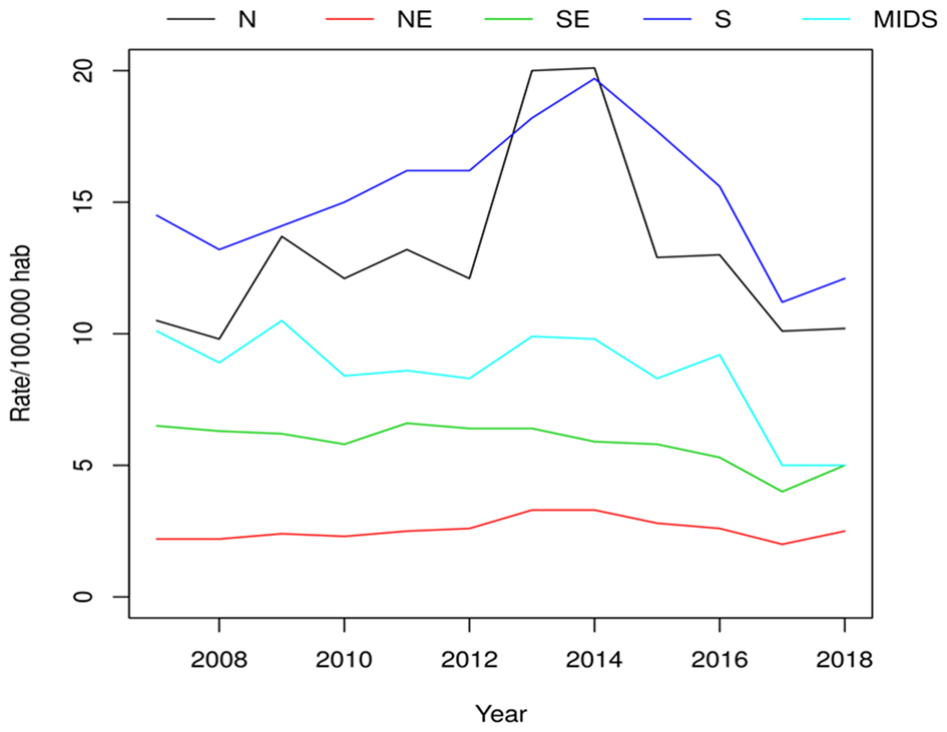
Hepatitis B infection rate per regions of Brazil from 2007 to 2018. The five regions of Brazil are represented as North (N), Northeast (NE), Midwest (MIDS), South (S) and southeast (SE). This figure was created by using the R software^19^ version 3.5.3 (https://www.R-project.org/).

**Figure 4:**
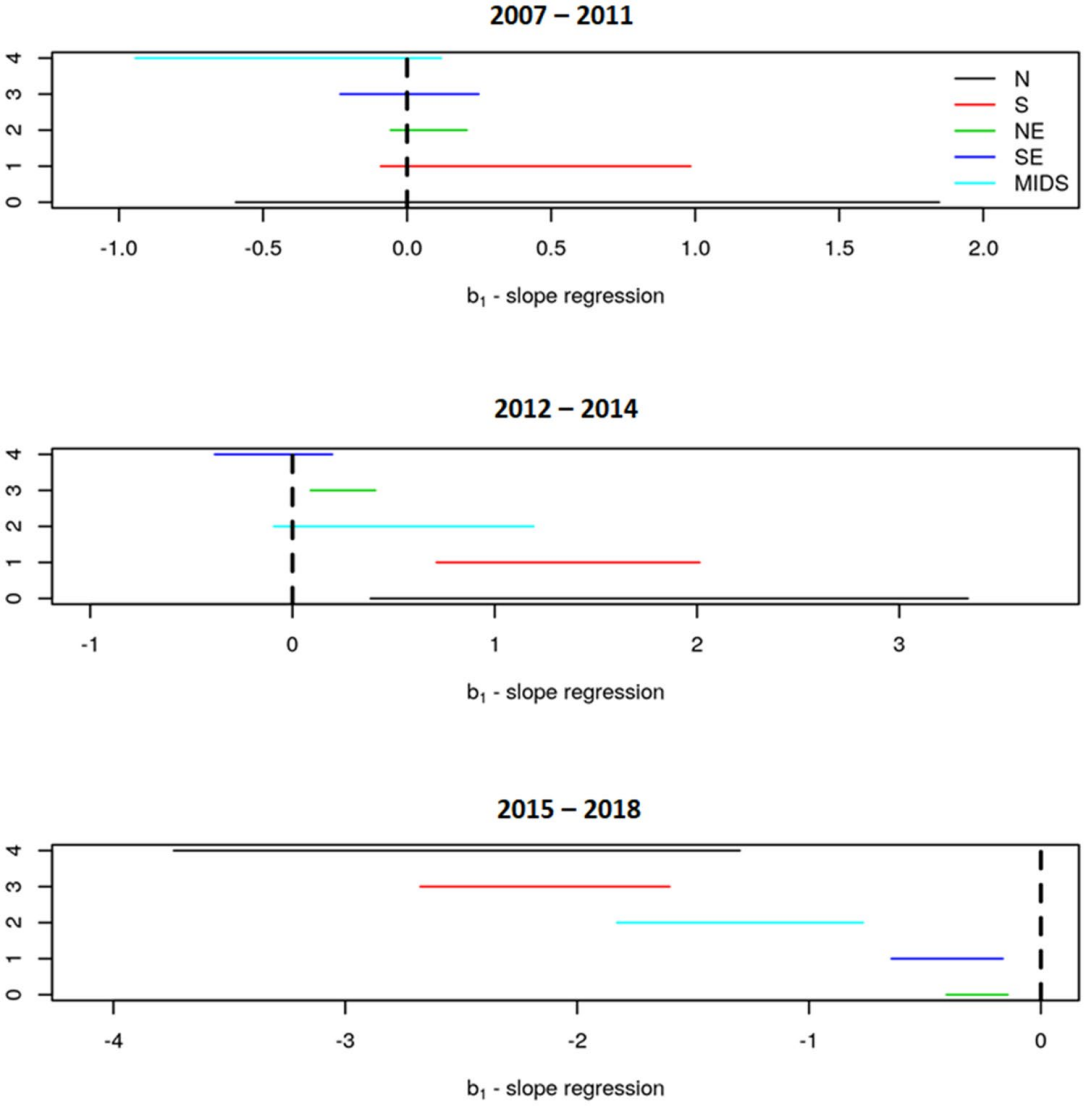
90% confidence interval for angular coefficients of Brazilian regions, from 2007 to 2011, 2012 to 2014, and 2015 to 2018. The five regions of Brazil are represented as North (N), Northeast (NE), Midwest (MIDS), South (S) and Southeast (SE). This figure was created by using the R software^19^ version 3.5.3 (https://www.R-project.org/).

Next, we evaluated temporal trends related to the HBV occurrence. The rate per 100,000 inhabitants with HBV in each region of Brazil is shown in Fig. 3. There was a high chance of the trend being significantly different between the periods 2007–2011, 2012–2014 and 2015–2018 (*P* < 0.05). During 2007–2011, the infection trend remained constant (Fig. 4). During 2012–2014, there was a significant increase in the notification of HBV infection in North, Northeast, and South regions (*P* = 0.05, *P* = 0.02, and *P* ≤ 0.0001 respectively). During 2015–2018, there was a reduction of HBV infection in all regions.

When analyzing trends related to the sources of infection over time, it was found that the behavioral characteristics varied (Table 5). Most sources of infection have shown a steady or decreasing trend over the last 10 years. Regarding the vertical transmission, there was an increasing from 2012 to 2014, which coincides with an improvement in the diagnostic of HBV due to the centralization of molecular biology tests and the expansion of rapid tests started in 2011^3^. No differences were observed for sex during 2007–2014 (*P* = 0.06), however, during 2015–2018, the trend between genders was significantly different (*P* = 0.01). A decreasing trend was observed for females and increasing for males.

**Table 5:** Suggestions of strategies as part of a multi-pronged approach to reducing burden of HBV infection considering the different regional realities in Brazil.

Region	Suggested strategy to prevent HBV infection
North	To prioritize actions and to address sexual transmission primarily for young people, such as expanding condom access and providing safe sex guidance
Northeast	To expand the educational actions for prevention and control of hepatitis B in young people
Midwest	To expand the educational actions for prevention and control of hepatitis B in young people
South	To encourage the adoption of good practices in health services, to reduce the risk of viral transmission especially for those over 40 years
	To encourage vaccination for adult population
	To strengthen actions to reduce vertical transmission To set up vaccination rooms in motherhood
Southeast	To improve the quality of Epidemiological Surveillance, especially regarding the proper completion of epidemiological investigation forms
	To encourage vaccination for adult population

**Figure 5:**
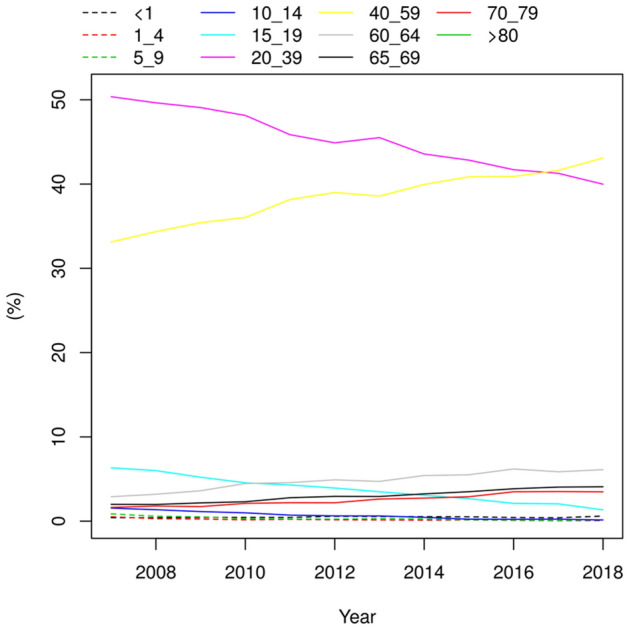
Hepatitis B infection trend according to age, distributed by year (from 2007 to 2018). The figure shows that individuals aged from 20 to 59 years old accounted for the higher number of HBV cases. Furthermore, it can be observed a decreasing of HBV cases for individuals aged 20–39 years old, while an increasing of HBV cases for individuals aged 40–59 years old during the years. Age (years), was categorized as < 1; 1–4; 5–9; 10–14; 15–19; 20–39; 40–59; 60–64; 65–69; 70–79; > 80. This figure was created by using the R software^19^ version 3.5.3 (https://www.R-project.org/).

It was also found an increasing trend in 40–59 years old individuals, and a decreasing trend in individuals under 39 years (Fig. 5). In summary, the trend analyzes demonstrated a statistically significant reduction (*P* < 0.05) in the HBV infection rate in all regions and most forms of transmission in the last 4 years. Additionally, there was an increasing trend of HBV infection in males and decreasing in females, as well as an increasing trend in individuals over 40 years old and decreasing in under 39 years old.

## Discussion

Here we described an observational, ecological analytical study to better understand the epidemiological scenario of HBV infection in different regions of Brazil during 2007–2018, and the historical trends associated with HBV infection throughout Brazil. Analysis of surveillance trends is an important way to guide better public health practices.

Interestingly, the Ministry of Health has successfully adopted control measures aiming to reduce the vertical transmission, such as compulsory childhood vaccination (initiated in 1998), rapid testing during prenatal healthcare visits, and the use of HBV immunoglobulin postpartum in babies born from HBV–carrying mothers^3^, which can explain the low proportion of the total cases of HBV infection in the 0–9 years old group.

Vieira and colleagues^22^ have previously reported that sexual contact is the main source of HBV infection in North region of Brazil. These differences can be related to different genotypes circulating around the country and to multi-factorial issues, few STI clinics, and high poverty, for example^23,24^. It is important to point out the need to improve the epidemiological surveillance in the Southeast region as data pertaining to presumptive source of infection (52.5%) were often not reported. This fact complicates the creation of effective preventive measures against HBV infection in the Southeast region.

A higher proportion (41.0%) was observed among adults (20–79 years old), which corroborates studies performed in Brazil and other countries^25–27^. The population among 20–39 years old are sexually active, being the sexual transmission the most important route for HBV infection^3^. Furthermore, it is important to note that there may be a reduced vaccine response in adults (over 40 years), especially men who are smokers or that presents co-morbidities^26,28^, which contribute to the risk factors associated with HBV infection keep increasing over time.

Although immunoglobulin and vaccine are freely available at public health facilities, vertical transmission still remains a prominent route of HBV transmission. Together, these strategies are able to prevent transmission in up to 95.0% of cases^1,3^. In the 20–39 years old age group, the most prominent presumptive source of infection was the sexual. Although strategies adopted by the Ministry of Health against HBV infection focus mainly on sexual practice, our findings suggested that this population are still practicing unprotected sex. We also observed that intravenous drug use and occupational accident were significantly associated with the 20–39 years old group. On the other hand, we were not able to link a single, prominent presumptive source of infection to the 40–59 years old age group due to gaps in the data (records being poorly recorded). This fact deserves further attention in order to identify potential sources of infection and perform effective measures to prevent HBV infection. Those who were ≥ 60 years old were more likely to be infected through surgical or dental treatment, transfusion, and hemodialysis.

Regarding the presumptive source of infection, no associations were observed for individuals from Northeast and Midwest regions. However, there was a strong association with the sexual contact for individuals from North region, which can also be corroborated by previous studies^22^, showing that sexual contact as the main source of HBV infection in that region. Individuals from the Southern region were more likely to be infected through transfusion, vertical transmission, surgical or dental treatment.

It is also important to point out the need to improve the epidemiological surveillance in the Southeast region once most of the available answers for clinical form of HBV infection (49.2%) and presumptive source of infection (59.1%) were not reported. This fact also highlights how difficult can be to adopt effective preventive measures against HBV infection in the Southeast region.

The profile of infected individuals differs greatly from one region to another. This fact reflects the diversity of public health in terms of genetic background, access to health information and health services, lifestyle, educational level, income, and other socialecnoomic status^29–37^. Hence, it is important to emphasize that national campaigns to prevent HBV infection would be unlikely to be effective unless it accounted for regional differences, including genetic background, access to health information and health services, lifestyle, educational level, and income. Table 4 presents some possible strategies that could be adopted considering the epidemiological profile of each region.

Educational actions should be recommended according to regional differences related to age, gender, and presumptive source of infection. The use social media could be relevant to facilitate the acceptance and spreading of the information. Moreover, HBV infection results in several outcomes from spontaneous cure, fulminant hepatitis, and chronic disease, which can also vary from asymptomatic carriers to cirrhosis. The laboratory follow-up is necessary in order to identify the need for specific therapeutic approaches. It would be also interesting if SINAN could establish HBV follow-up bulletins similar to what is currently used in Brazil with some chronic conditions such as Hansen’s disease and Tuberculosis^38,39^.

Due to the complexity of HBV infection, complementary actions in partnership with organized civil society, NGOs, mental health care network, and social care services should be considered. The health services such as the surveillance systems and health care units could work together, reinforcing the importance of adequate fulfillment of notifications and investigations, closely monitoring the active cases, perform active search for absentees, expand immunization campaigns, and promote educational actions. Indeed, as pointed out by Melo et al.^40^, underreporting of notifications is a reality experienced in patient care units. The main difficulties in the notification process seems to be linked to the conduct of the healthcare professionals, which have difficulties in diagnosing cases, lack of an adequate report, and delay in the notification (which can lead to loss of notification)^40^. Besides the unavailability of registers to record patient information in standard manner, the lack of improved data quality and completeness, quality-enhanced data entry are such challenges for developing and conducting observational studies using secondary data collected from notification systems, showing a negative impact on data accuracy^41–43^. Furthermore, capturing data in different tools with different formats, as well as not capturing and transferring the data properly could cause variations in data quality and lead to estimates not consistent with the reality of different population.

On the other hand, using secondary data is important to put forward preventive and control strategies through exploring epidemiological questions in different sub-populations, performing longitudinal studies at relatively lower cost by using retrospective data, and help answering questions that require detailed data on hard-to-reach populations^13,44^.

Campaigns for testing and implantation of HBV and HCV diagnosis started by the Ministry of Health since 2005, and the national distribution of rapid tests started in 2011 facilitated the access of the population to HBV testing. However, it was not able to reduce the number of infections^3^. Indeed, there was no significant association for the Southeast and Midwest regions during that period, and although strategies adopted by the Ministry of Health are national, in the short period, there were no equal results for all regions.

Medium- and long-term actions, such as the progressive expansion of access to immunization and better access to diagnostics and treatment, had positively impacted in a decreasing of HBV infection in all regions of Brazil. HBV has tended to decline in other countries, but remains more prevalent than other viral infections that have similar transmission mechanisms, such as HIV^20^. This fact could be related to intrinsic characteristics of HBV itself, such as higher infectivity and greater resistance in the environment^45,46^. Moreover, immunization campaigns were performed only for individuals under 20 years of age and more vulnerable populations. It is also important to emphasize that the vaccine has been implemented to the basic calendar of these individuals recently^1,3^ and the adult population traditionally does not have the habit of getting vaccinated.

Our study has limitations. Biases could have been introduced owing to incompleteness of data or data errors. Surprisingly, as an example, Table 2 shows that 30% of individuals 0–9 years old were infected through sexual intercourse, as well as 2.4% of this age group were intravenous drug users. These findings are concerning and merit further investigation for validity. This information raises concerns about the reliability of the data included into the SINAN database, suggesting that major errors could be introduced and need clear attention. Although SINAN is a very robust database, it is essential to encourage the correct and accurate filling out data into the SINAN system to generate reliable information once the use of databases is very useful to improve the quality of care and to contribute with specific preventive strategies. Furthermore, the analysis of factors such as vaccination history or coverage would be very helpful to rule out some bias that could potentially be introduced in the study. However, we were not able to perform assess vaccination history or coverage and perform further analysis to verify its impact on transmission factors more likely to be associated with HBV infection. Future studies, including meta-analysis and casual inference under the Mendelian Randomization analysis framework, would help to better clarify the specific profile of HBV infections in Brazil^47–51^.

Finally, it is important to highlight that the use of secondary databases has many advantages: the analyses are fast and less expensive way to acquire relevant information, and there is a possibility to conduct a temporal follow-up and to have large volume of information with population and geographic amplitude.

## Conclusions

Our findings suggest that, in Brazil, HBV infection seems to be more prevalent in 20–39-year-old males, with sexual transmission being the main presumptive source of infection. Hence, specific preventive measures should be addressed to men and 40 years old individuals due to the increasing trend of HBV infection. The profile of HBV infection differs significantly between regions, reinforcing that specific surveillance and control strategies are required for each reality. Finally, there is an ongoing need to strengthen surveillance proficiency, with the aim of reducing incompleteness and errors in data, as well as a need to strengthen efforts to incorporate findings from surveillance into routine HBV prevention programs.

## Supplementary Information

Supplementary Information 1.

Supplementary Information 2.

Supplementary Information 3.
