# Supplementary material for: Clinical, epidemiological aspects, and trends of Hepatitis B in Brazil from 2007 to 2018
Source: Sci Rep. 2021 Jul 7;11:13986. doi: 10.1038/s41598-021-93434-y (PMC8263714; doi:10.1038/s41598-021-93434-y)
Supplement: Supplementary file 1 — Supplementary Information 1. [file 41598_2021_93434_MOESM1_ESM.doc]

Supplementary table 1 – Distribution of the 172,090 Hepatitis B virus cases per region of Brazil during 2007–2018.

| **Region** | **Total** | **2007** | **2008** | **2009** | **2010** | **2011** | **2012** | **2013** | **2014** | **2015** | **2016** | **2017** | **2018** |
| --- | --- | --- | --- | --- | --- | --- | --- | --- | --- | --- | --- | --- | --- |
| North | 27121 | 1545 | 1545 | 1545 | 1545 | 1545 | 1545 | 1545 | 1545 | 1545 | 1545 | 1545 | 1545 |
| Northeast | 17813 | 1120 | 1120 | 1120 | 1120 | 1120 | 1120 | 1120 | 1120 | 1120 | 1120 | 1120 | 1120 |
| Midwest | 15453 | 1340 | 1340 | 1340 | 1340 | 1340 | 1340 | 1340 | 1340 | 1340 | 1340 | 1340 | 1340 |
| South | 53373 | 3884 | 3884 | 3884 | 3884 | 3884 | 3884 | 3884 | 3884 | 3884 | 3884 | 3884 | 3884 |
| Southeast | 58330 | 5089 | 5089 | 5089 | 5089 | 5089 | 5089 | 5089 | 5089 | 5089 | 5089 | 5089 | 5089 |
