# Supplementary material for: Clinical, epidemiological aspects, and trends of Hepatitis B in Brazil from 2007 to 2018
Source: Sci Rep. 2021 Jul 7;11:13986. doi: 10.1038/s41598-021-93434-y (PMC8263714; doi:10.1038/s41598-021-93434-y)
Supplement: Supplementary file 2 — Supplementary Information 2. [file 41598_2021_93434_MOESM2_ESM.doc]

Supplementary table 2 – Distribution of different clinical forms of Hepatitis B virus infection in Brazil during 2007–2018.

| **Clinical form of HBV infection*** | **Total** | **2007** | **2008** | **2009** | **2010** | **2011** | **2012** | **2013** | **2014** | **2015** | **2016** | **2017** | **2018** |
| --- | --- | --- | --- | --- | --- | --- | --- | --- | --- | --- | --- | --- | --- |
| Acute | 22498 | 2104 | 2015 | 2109 | 1825 | 1844 | 1888 | 2184 | 2032 | 1675 | 1730 | 1655 | 1437 |
| Chronic | 139132 | 10217 | 9831 | 10994 | 10614 | 11951 | 11752 | 14289 | 14661 | 12784 | 12178 | 11288 | 8573 |
| Fulminant | 289 | 28 | 44 | 33 | 25 | 19 | 20 | 25 | 21 | 15 | 21 | 22 | 16 |
| Inconclusive diagnosis | 6793 | 495 | 508 | 462 | 486 | 480 | 451 | 749 | 713 | 765 | 649 | 593 | 442 |
| Missing data | 3378 | 134 | 196 | 242 | 218 | 280 | 272 | 354 | 300 | 329 | 397 | 365 | 291 |

*Information regarding asymptomatic cases were not available
