# Supplementary material for: Clinical, epidemiological aspects, and trends of Hepatitis B in Brazil from 2007 to 2018
Source: Sci Rep. 2021 Jul 7;11:13986. doi: 10.1038/s41598-021-93434-y (PMC8263714; doi:10.1038/s41598-021-93434-y)
Supplement: Supplementary file 3 — Supplementary Information 3. [file 41598_2021_93434_MOESM3_ESM.doc]

Supplementary table 3 – Number of missing data related to Hepatitis B virus infection according to different variables in Brazil during 2007–2018.

| **Variables** | **Total** | **2007** | **2008** | **2009** | **2010** | **2011** | **2012** | **2013** | **2014** | **2015** | **2016** | **2017** | **2018** |
| --- | --- | --- | --- | --- | --- | --- | --- | --- | --- | --- | --- | --- | --- |
| Gender | 25 | 1 | 2 | 2 | 2 | 3 | 4 | 3 | 1 | 1 | 1 | 1 | 4 |
| Age (years) | 33* | 4 | 3 | 6 | 7 | 4 | 3 | 3 | 3 | NA | NA | NA | NA |
| Education | 50976 | 3684 | 3684 | 3684 | 3684 | 3684 | 3684 | 3684 | 3684 | 3684 | 3684 | 3684 | 3684 |
| Presumptive source of infection | 95093 | 7050 | 6898 | 7465 | 7278 | 8018 | 7804 | 9515 | 9631 | 8657 | 8503 | 8005 | 6269 |
| Clinical form of HBV infection | 3378 | 134 | 196 | 242 | 218 | 280 | 272 | 354 | 300 | 329 | 397 | 365 | 291 |

*The total might not be equal to 33 due to the lack of information on missing data for 2015, 2016, 2017, and 2018

NA – not available
